# Temporal order of bipolar cell genesis in the neural retina

**DOI:** 10.1186/1749-8104-3-2

**Published:** 2008-01-23

**Authors:** Eric M Morrow, C-M Amy Chen, Constance L Cepko

**Affiliations:** 1 Division of Genetics, New Research Building, 77 Avenue Louis Pasteur, Children's Hospital Boston, Harvard Medical School, Boston, MA 02115, USA; 2 Department of Psychiatry, 15 Parkman Street, Massachusetts General Hospital, Harvard Medical School, Boston, MA 02114, USA; 3 Novartis Institute for Biomedical Research, 500 Technology Square, Cambridge, MA 02139, USA; 4 Department of Genetics and Howard Hughes Medical Institute, New Research Building, 77 Avenue Louis Pasteur, Harvard Medical School, Avenue Louis Pasteur, Boston, MA 02115, USA

## Abstract

**Background:**

Retinal bipolar cells comprise a diverse group of neurons. Cone bipolar cells and rod bipolar cells are so named for their connections with cone and rod photoreceptors, respectively. Morphological criteria have been established that distinguish nine types of cone bipolar cells and one type of rod bipolar cell in mouse and rat. While anatomical and physiological aspects of bipolar types have been actively studied, little is known about the sequence of events that leads to bipolar cell type specification and the potential relationship this process may have with synapse formation in the outer plexiform layer. In this study, we have examined the birth order of rod and cone bipolar cells in the developing mouse and rat *in vivo*.

**Results:**

Using retroviral lineage analysis with the histochemical marker alkaline phosphatase, the percentage of cone and rod bipolar cells born on postnatal day 0 (P0), P4, and P6 were determined, based upon the well characterized morphology of these cells in the adult rat retina. In this *in vivo *experiment, we have demonstrated that cone bipolar genesis clearly precedes rod bipolar genesis. In addition, in the postnatal mouse retina, using a combination of tritiated-thymidine birthdating and immunohistochemistry to distinguish bipolar types, we have similarly found that cone bipolar genesis precedes rod bipolar genesis. The tritiated-thymidine birthdating studies also included quantification of the birth of all postnatally generated retinal cell types in the mouse.

**Conclusion:**

Using two independent *in vivo *methodologies in rat and mouse retina, we have demonstrated that there are distinct waves of genesis of the two major bipolar cell types, with cone bipolar genesis preceding rod bipolar genesis. These waves of bipolar genesis correspond to the order of genesis of the presynaptic photoreceptor cell types.

## Background

The retina offers an excellent model system for dissecting the mechanism of neural development in vertebrates [1-3]. The adult retina contains a complement of well-characterized neurons and glia in three cellular layers (outer nuclear, inner nuclear and ganglion cell layers) separated by two distinct plexiform layers (the inner and outer plexiform layers) containing cellular processes and synapses [4]. The inner plexiform layer (IPL) contains bipolar-ganglion cell connections, as well as modulatory amacrine interneuron synapses. The outer plexiform layer (OPL) contains the tripartite ribbon synapses of presynaptic horizontal and photoreceptor cells and the post-synaptic bipolar cells. Given the well characterized cellular morphology and biochemistry of the retina, the developmental processes of neurogenesis, cell fate determination, neuronal and glial differentiation have been actively studied. Bipolar cell type specification and its potential relationship with synaptogenesis have been relatively less well examined [5,6].

**Figure 1:**
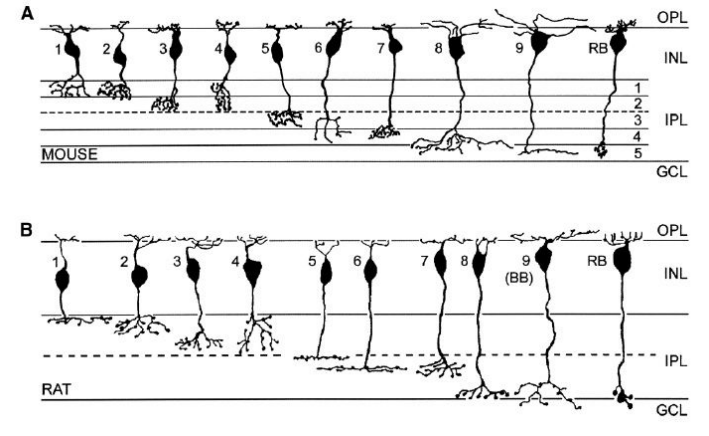
Morphological subtyping of bipolar cells in mouse and rat. Adapted from Ghosh *et al*. [8]. Shown are drawings of bipolar cells after injection with intracellular dyes. The cone bipolars are numbered 1 through 9, and the rod biolar is labeled RB. The layers of the retina are the outer plexiform layer (OPL), inner nuclear layer (INL), the inner plexiform layer (IPL) and the ganglion cell layer (GCL). The dashed line midway through the IPL represents the division above which the axons of OFF-bipolars extend and below which axons of ON-bipolars are found.

Retinal neurons demonstrate extensive diversity. For example, based on morphological criteria at least 22 distinct types of amacrine cells have been described [7]. Bipolar cells have been classified based on multiple criteria [8]. For example, classification based on the presynaptic photoreceptor cell type divides bipolars into two broad classes, namely the cone bipolar cells and rod bipolar cells. In rodents, rod and cone ribbon synapses are morphologically distinct [9]. Further, bipolar cells are also classified based on morphology, biochemistry, neurochemistry and functional criteria. Based on morphology, there are nine distinct cone bipolar cells and one rod bipolar (Figure 1) [8]. One example of distinct biochemistry or protein expression is that of protein kinase C (PKC) alpha, which is expressed in rod bipolar cells but not in cone bipolar cells [10]. Finally, bipolar cells are classified based on functional criteria, that is, ON-bipolar cells and OFF-bipolar cells. ON-bipolar cells are depolarized by the light response in photoreceptor cells and have processes that end in the inner half of the IPL. Rod bipolar cells are exclusively ON-bipolars. OFF-bipolar cells are hyperpolarized by the light response in cones and have processes that end in the outer half of the IPL [11].

Little is known about the mechanism of bipolar cell specification in the neural retina. In rodents, with respect to bipolar type specification, this is in part due to an incomplete description of the sequence of events during bipolar differentiation. In this study, we describe the kinetics of genesis of bipolar cell types in both the mouse and rat retina *in vivo*. We have labeled bipolar cells during the terminal mitosis and subsequently studied the relative genesis of cone and rod bipolar cells during the first postnatal week. Both morphology and immunohistochemistry were used to identify types of bipolar cells. We directly demonstrate that there is a clear temporal relationship to bipolar type genesis, with birth of cone bipolar cells distinctly preceding that of rod bipolar cells both in both mouse and rat. This study represents the first example of a description of the kinetics of the genesis of bipolar cell types and contributes to the descriptive framework through which developmental models of cell type specification may be tested.

## Results

**Figure 2:**
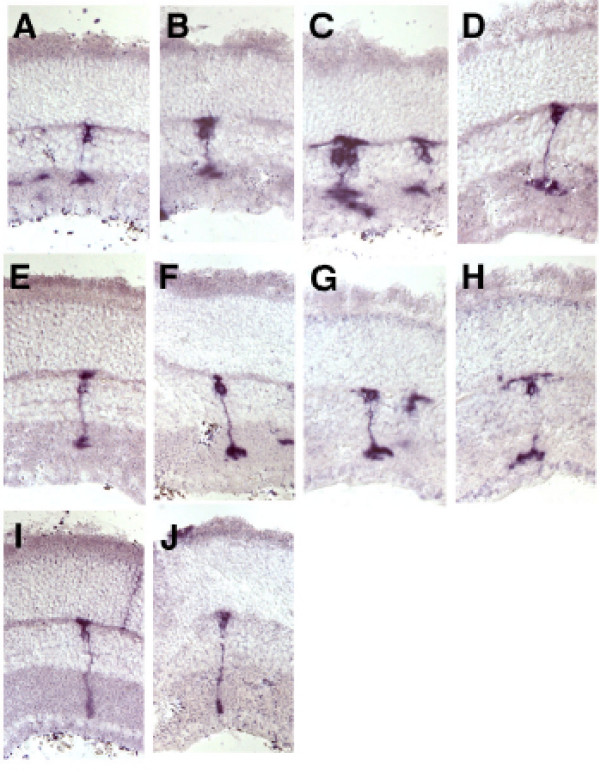
Bipolar cell morphology observed using retroviral lineage analysis *in vivo*. The retroviral vector Ely expresses the human placental alkaline phosphatase as a histochemical reporter. Neonatal rat pups were injected *in vivo *during development at PO, P4, and P6 and sacrificed at P21 after retinal differentiation is complete. Cryosections were made and the type of cell in each clone was assessed using morphological criteria. **(a-h) **Cone bipolar cells wherein the process ends prior to the innermost aspect of the IPL. **(i, j) **Rod bipolar cells whereby processes extend to the innermost aspect of the IPL and display narrow endings and characteristic dendrites.

**Figure 3:**
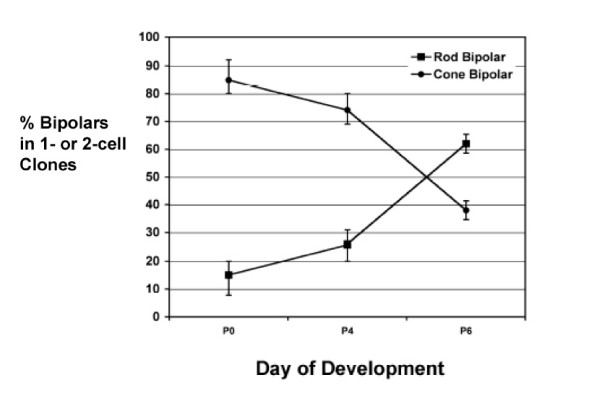
Bipolar birthdating in the rat retina *in vivo *using retroviral lineage analysis. Bipolar cells in clones from pEly control virus infections that contained one or two cells (usually rod-bipolar or bipolar-only clones) were scored. Bipolar cells in these clones were interpreted to be born on the day of infection. The percentage of cone bipolar cells and rod bipolar cells interpreted to be born on P0, P4 and P6 are shown. Retinas were infected on the indicated day. For P0, six retinas were used. For P4, three retinas were used. For P6, two retinas were used. A total of 200 clones were scored for each time point.

Birth order of rod bipolar and cone bipolar cells in the normal retina was determined by two methods: retroviral lineage tracing in rat and classical birthdating using tritiated-thymidine in mouse. In the retroviral lineage study, clones resulting from retroviral transduction with a replication-incompetent retrovirus expressing the histochemical enzyme alkaline phosphatase were examined for the ratios of rod bipolar and cone bipolar cells. Dividing progenitor cells were transduced with the retrovirus ELY by *in vivo *retinal injections on postnatal day 0 (P0), P4 and P6. Bipolar cells within one or two cell clones (that is, most often bipolar-only or rod-bipolar clones) were scored in adult retina (after P21) for rod bipolar or cone bipolar morphology (Figures 1 and 2). Small clones (one or two cells) comprise cells that were generated shortly after the viral infection. This is deduced from the fact that the virus integrates into the M phase chromatin of a mitotic cell, and is inherited by only one daughter of the first cell cycle following infection [12]. Subsequent cell cycles lead to inheritance of the viral DNA by all daughters. A one cell clone occurs if the viral DNA integrated into the chromatin that was partitioned into a daughter cell that exited cell cycle just after the first M phase following infection. A two cell clone would occur if the viral DNA integrated into a daughter that went on to make two postmitotic daughters in the next cell cycle. Since the cell cycle is approximately 24 hours at P0 [13,14], one can estimate that one cell clones and two cell clones comprise cells born on P1-P2 if the virus was delivered on P0. These data provide a method for determining birthdate of rod bipolar and cone bipolar cells. As shown in Figure 3, 85% of bipolar cells in clones of < 3 cells derived from infection at P0 were cone bipolar cells. The percentage of cone bipolar cells decreased to 74% when retinal progenitor cells were infected at P4. This ratio reversed when the infection was at P6, with 72% of the bipolar cells being rod bipolar cells and 38% being cone bipolar cells.

**Figure 4:**
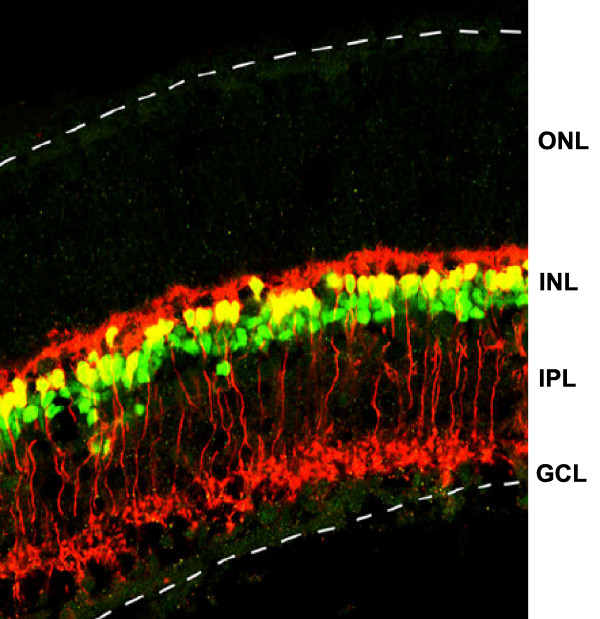
Immunohistochemical staining of rod and cone bipolars in the mouse retina in tissue section. Rod bipolars are double stained with Chx10 and PKC antibodies, while cone bipolars are positive for Chx10 staining in the nuclei but are not stained by PKC antibodies. Confocal images of P21 mouse retinal sections stained with Chx10 antibody (green nuclear stain) and PKC antibodies (red staining throughout bipolar processes). The margins of the retina are indicated with dashed lines. Rod bipolars are double labeled (yellow nuclei and red processes that extend into the inner half of the IPL and characteristic dendritic process). Cone bipolars are labeled by only Chx antibodies (green nuclei in the INL). GCL, ganglion cell layer; INL, inner nuclear layer; IPL, inner plexiform layer; OPL, outer plexiform layer.

**Figure 5:**
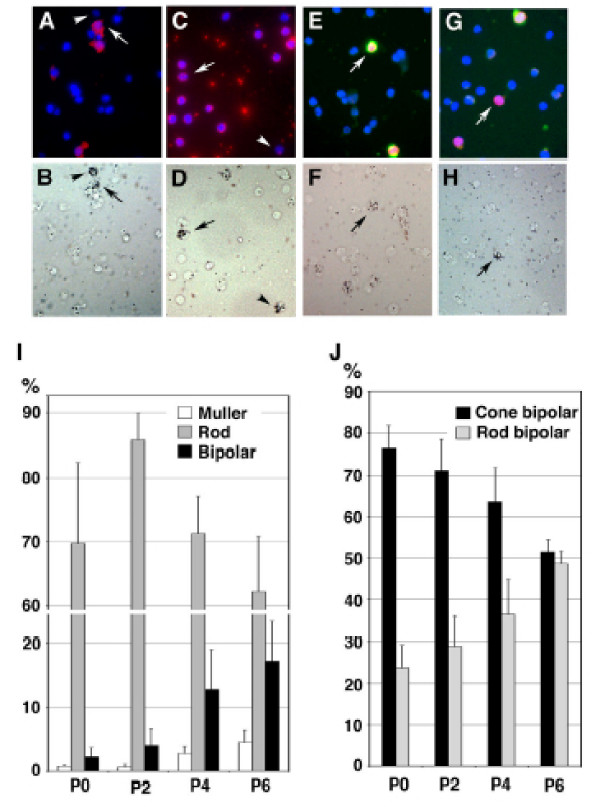
Bipolar birthdating in the postnatal mouse retina using combined tritiated-thymidine and immunofluorescence to determine bipolar type. Immunofluorescent staining of dissociated P16 retinas with **(a) **anti-glutamine synthetase (Muller glia), **(c) **Rho4D2 (rods), **(e) **anti-Chx10 and anti-PKC-positive (yellow; rod bipolar cells), and **(g) **anti-Chx10 and and anti-PKC-negative (red; cone bipolar cells). Blue nuclear stain is DAPI. The arrow in (e) shows a triple stained rod bipolar with blue (DAPI) nuclei, also stained with Chx10 (red), leaving a purple nuclei, and also with PKC+ (green) processes. The arrow in (g) shows a cone bipolar cell with a purple nucleus stained only for DAPI (blue) and Chx10 (red), and PKC-negative processes. **(b, d, f, h) **Autoradiography of [3H]thymidine from the same fields show birthdated cells, which have a high density of silver grains (arrows and arrowheads). Birthdated Muller glia are indicated by the arrow in (a, b), non-Muller glia by the arrowhead in (a, b), rod by the arrow in (c, d), non-rod by the arrowhead in (c, d), rod bipolar cell by the arrow in (e, f), and cone bipolar cell by the arrow in (g, h). **(i) **The percentage of heavily labeled cells of the indicated cell type (that is, interpreted as cells born) on P0, P2, P4 and P6 was scored on each retina. **(j) **The percentage of rod versus cone bipolar type was further divided. P0, n = 3 retinas; P2, n = 8 retinas; P4, n = 6 retinas; and P6, n = 5 retinas.

To confirm that cone bipolar cells are born prior to rod bipolar cells, a classical birthdating experiment was carried out for the mouse. The birthdays of rods, Muller glia, cone bipolar and rod bipolar cells were determined following injection with a single dose of [^3^H]-thymidine at P0, P2, P4 or P6. Retinas were dissected and dissociated on P16 after bipolar cell differentiation is complete. Immunocytochemistry was performed with anti-rhodopsin, a photoreceptor marker, anti-glutamine synthetase, a Muller glial marker, anti-Chx10, a bipolar marker, and anti-PKCα, a rod bipolar marker, followed by autoradiography. Retinal cells exhibiting more than one-half of the maximum number of silver grains were scored as born within one day of the [^3^H]-thymidine injection [15]. In order to confirm bipolar type, we double-stained adult retinas with the antibodies against Chx10 and PKCα (Figure 4). Consistent with our experience and the literature [16], we found that anti-Chx10 antibodies stained all bipolar nuclei (Figure 4, green), and anti-PKC stained only rod bipolars including processes that extended into the inner half of the IPL and demonstrated morphology consistent with rod bipolars (Figures 1 and 4). Thereby, cells expressing both Chx10 and PKCα (Figures 4 and 5j, yellow) were scored as rod bipolar cells, and cells expressing Chx10 but not PKCα (Figures 4 and 5j, green) were scored as cone bipolar cells. Although some retinal cells that are Chx10+ and PKCα- are Muller glia, we have found that it is very difficult to detect the Chx10 immunocytochemical signal in Muller glia [17]. Thus the number of cells scored as bipolar cells that are actually Mulller glia should be very small. After dissociation of adult retina, 40.94 ± 3.87% of Chx10+ cells were positive for PKC (that is, rod bipolar cells) and 59.06 ± 3.87 % of Chx10+ cells were PKC negative (that is, cone bipolar cells), which is generally consistent with what was observed in section (Figure 4) and other reported quantification [18]. Examples of birthdated Muller glia, rod photoreceptors, rod bipolar cells and cone bipolar cells are shown in Figure 5.

The great majority of cells generated postnatally were rod photoreceptor cells (Figure 5, Table 1), as determined previously [19], comprising 69.7%, 85.7%, 71.2% and 62.7% of the cells born on P0, P2, P4, and P6, respectively. Muller glial cells were a small percentage of the retinal cells, representing 0.7%, 0.6%, 2.7% and 4.0% of the cells born on P0, P2, P4, and P6, respectively. Bipolar cells were born at an increasing rate during this period: 2.2%, 4.0%, 12.8% and 16.0% of the birthdated population on P0, P2, P4, and P6, respectively (Figure 5i, Table 1). Of all bipolar cells born, cone bipolar cells comprised 76.2% at P0, 71.1% on P2, 63.5% on P4 and 51.6% on P6 (Figure 5j). These birthdating data in the mouse are consistent with the observations of the retroviral data in the rat, demonstrating that cone bipolar cells are born before rod bipolar cells (Figure 3).

**Table 1:** Postnatal retinal cell genesis as quantified by thymidine birthdating and either immunofluorescent or histological identification of cell fate

	P0		P2		P4		P6	
				
Cell type	IF	Hist*	IF	Hist*	IF	Hist*	IF	Hist*
Bipolar	2.19 ± 1.45	6.51	3.98 ± 2.66	17.04	12.81 ± 6.16	31.81	16.01 ± 6.31	25.74
Cone type	1.67 ± 1.15	NA	2.73 ± 1.90	NA	7.96 ± 3.23	NA	8.20 ± 2.97	NA
Rod type	0.52 ± 0.32	NA	1.21 ± 1.01	NA	4.85 ± 3.35	NA	7.82 ± 3.41	NA
Amacrine	NA	9.16	NA	0.05	NA	0.00	NA	0.00
Rod	69.69 ± 12.65	81.67	85.73 ± 4.15	79.51	71.22 ± 5.88	59.54	62.74 ± 8.63	60.59
Muller	0.68 ± 0.26	2.66	0.58 ± 0.43	3.40	2.73 ± 1.08	8.65	4.04 ± 1.97	13.67

Postnatal mice were injected with tritiated thymidine at the developmental time indicated. For the immunofluorescent (IF) methodology, retinae were harvested at P16, dissociated and stained using established cell-type specific markers, namely anti-Chx10 (bipolar cells), Rho4D2 (rod photoreceptors), and anti-glutamine synthetase (Muller cells). *For the histochemical (Hist) estimates all data are based on [19]. Cells born from the central and peripheral sections were summed to thereafter derive a percentage of each cell type

## Discussion

Retinal bipolar cells exhibit significant diversity, categorized by biochemical, morphological and physiological properties. In the present study, we addressed the following question directly: is there an order of genesis of bipolar cell types? The answer to this question was not discoverable by classic tritiated-thymidine birthdating studies due to the requirement for morphologic or immunohistochemical markers in order to distinguish bipolar types [19-23]. Using two independent *in vivo *methods, we demonstrate that there is a distinct order of genesis of bipolar types. Using combined tritiated-thymidine and immunohistochemistry in mouse, and retroviral transduction in rat, we have found that the genesis of cone bipolar cells definitively precedes the genesis of rod bipolar cells.

Photoreceptors are well known to be generated in two distinct waves [19-23]. Across many species, cone photoreceptors are generated first. In rats and mice, cone genesis occurs during embryonic retinal development and is largely completed prior to bipolar cell genesis [19,20,23]. Rod genesis peaks at birth, and bipolar genesis begins around birth, with a peak during the first postnatal week. Here we show that bipolar cell types also appear to be specified in two distinct, but overlapping, waves. There is a striking correspondence of the waves of bipolar cell specification with the waves of genesis of the presynaptic photoreceptor. Cone photoreceptors are generated early during retinal development and begin to express a subset of markers during embryonic development [24-26], thereby likely reflecting early differentiation, that is, preceding bipolar genesis and differentiation. As cone differentiation precedes bipolar differentiation, these data allow a model whereby the presynaptic cone induces the postsynaptic bipolar to terminally differentiate as a cone bipolar. In a study of bipolar synapse formation in both OPL and IPL, the glutamatergic vesicular transporter VGLUT1 was first detected in cone photoreceptor terminals at P2, several days before initiation of cone ribbon synapses in the OPL at P4-5, and preceding the later appearance of rod spherules in the OPL [27]. Additional studies have confirmed the early, first appearance of cone photoreceptor synaptogenesis in the OPL preceding that of rods [28,29].

These studies, combined with the bipolar birthdating data presented here, are consistent with the possibility that cones may induce specification and/or differentiation of cone bipolar cells. Indeed this may be an early event in retinal circuit formation in the OPL; however, existing functional data may not fully support this model. At present, there are few mouse models where cone photoreceptors have been disrupted and subsequent bipolar differentiation has been examined in detail. In the example of the retina-restricted Otx2 knock-out, cone and rod photoreceptors fail to develop [30]. In these retina, Chx10-positve cells are noted to be dispersed across the retina. However, analysis of bipolar type and OPL development have not been reported, though expression of at least one bipolar cell marker, PKCα, is still present. As this marker can also be expressed by a type of amacrine cell, it is not clear if this reflects expression in rod bipolar cells. Further, we do not favor the hypothesis that rod photoreceptors induce rod bipolars, as there is an absence of rods in the Nrl mutant retina, yet the level of expression of rod bipolar markers appears to be similar to those found in wild-type retinas [31]. Overall, the data presented here draw attention to the need for further experiments testing the relationships between the development of synapses in the OPL with bipolar cell type specification.

Finally, the data presented here also provide a quantitative analysis of postnatal retinal cell genesis that is distinct from the one classic study on this topic [19]. In the classic study, cell type is determined by position of heavily labeled nuclei in retinal sections, whereas in this study, we employed immunocytochemistry with established cell type-specific markers. Further, the quantification in the study by Young relies on counting all heavily labeled cells in samples of sections from the central and peripheral retina. Here, we achieve whole retina sampling by quantification on dissociated cell preparations from adult retina. As shown in Table 1, while the relative timing of the genesis of major cell types is consistent, Young's study reports a higher percentage of INL cell types at each developmental time in lieu of rod photoreceptors. We believe that this distinction is most likely due to the difference in sampling, that is, finite sampling of sections in the classic method versus a complete retina averaging from the dissociated cell/immunohistochemical method. For example, at the P6 developmental time, the measures are based entirely on cells from the peripheral sections as histogenesis was reported complete in the central retinal sample [19].

## Conclusion

Cone bipolar genesis precedes that of rod bipolar genesis. The order of specification of bipolar cell types therefore mimics the order of genesis of the presynaptic photoreceptors. As several studies have demonstrated that cone differentiation and synaptogenesis precedes that of rods, as well as bipolar differentiation [27-29], these data together are consistent with a model wherein interactions between bipolars and cones in the OPL may play a role in at least leading cone bipolar terminal differentiation. Future studies, specifically those examining bipolar differentiation in mutants that disrupt formation of cone photoreceptors, will be necessary to further dissect the mechanisms used for bipolar cell specification and synapse formation in the OPL. In addition to elucidating basic mechanisms of neural development, these future studies may have importance to understanding the potential for tissue transplantation and regenerative therapy in retinal degenerative disease. In these diseases, transplanted neural progenitors would be required not only to undergo appropriate photoreceptor differentiation, but also to integrate into functional retinal circuitry.

## Materials and methods

### Animals

Timed pregnant rats (Sprague-Dawley) and mice (C57/B6) were acquired from either Taconic Farms, Germantown, NY, USA or Charles River, Wilmington, MA, USA and handled in accordance with IACUC guidelines and Harvard Center for Animal Resources and Comparative Medicine.

### Plasmids

pELY is a retroviral vector modified from the pLIA vector [32]. The 5' portion of the retroviral *gag *gene, with its translation initiation codon mutated, is present within pLIA, creating a 5' untranslated region that is the same as the gag gene mRNA. In pELY, the *gag *gene was completely deleted, creating a shortened 5' untranslated region, which may increase the expression level. pELY was also modified to carry multiple cloning sites (*Cla*I/*Bst*BI/*Sna*BI/*Xho*I/*Eco*RI) to facilitate the cloning process.

Bacterial expression plasmids containing GST fusion of mouse Chx10 amino-terminal and carboxy-terminal fragments were constructed as following: The *Eco*RI/*Xho*I fragments of amino-terminal and carboxy-terminal regions of mouse Chx10 were PCR amplified using GAGAATTCCGGGAGATGACGGGGAAAGC and TCGAGTCACTTGCTCTGGTTTAAAGCCG primer pairs and GAGAATTCCTGGAGGCAGCAGCTGAGTC and TTCTCGAGGCCCTAAGCCATGTCCTC primer pairs and subcloned into the *Eco*RI/*Xho*I site of the pGEX4T1 vector to generate pGEXmChx10N and pGEXmChx10C, respectively.

### Retroviral production and clonal analysis

High titer retroviral stocks were produced in the ecotropic producer cell line (Phoenix-E) and viral titer was determined on NIH-3T3 cells. *In vivo *infection in retina and clonal analysis were done as described previously [33].

### Generation of Chx10 antiserum

GST fusion constructs containing the mouse Chx10 amino-terminal domain and carboxy-terminal domain, pGEXmChx10N and pGEXmChx10C, were expressed in *Escherichia coli*. The fusion proteins were affinity purified using glutathione beads and injected into rabbits as a mixture to raise Chx10 antiserum.

### Immunohistochemistry

Immunostaining of retinal cryosections or dissociated cells were performed as described previously [34]. The primary antibodies for staining sections were used at the following dilution: Chx10 antiserum, 1:2,000; PKCα, 1:100 (Oncogene Science, Cambridge, MA, USA); glutamine synthetase, 1:500 (Chemicon, Billerica, MA, USA); Rho4D2, 1: 100 [35].

### Birthdating

Mouse pups were injected intraperitoneally with a single dose of [3H]-thymidine according to their body weight (10 μCi per gram of body weight). In general, 15 μCi were injected to each P0 pup, 20 μCi to each P2 pup, 25–30 μCi to each P4 pup, and 40 μCi to each P6 pup. Retinas were dissected at P16 and dissociated with papain as described previously [34]. Antibody staining described above and autoradiography were carried out as described previously [15].

### Retinal dissociation

Neural retinae were dissected free of other ocular tissues and incubated for 10 minutes at room temperature in Hank's Buffered Salt Solution (HBSS) lacking Ca^2+^/Mg^2+ ^(Life Technologies, Gaithersburg, MD, USA) to which trypsin (Worthington, Freehold, NJ, USA) was added to a final concentration of 1 mg/ml. After trypsinization, soybean trypsin inhibitor (Sigma, St Louis, MO, USA) was added to a final concentration of 2 mg/ml. The cells were then pelleted by centrifugation (1,200 rpm, 5 minutes), resuspended, and gently triturated to a single cell suspension in HBSS containing 100 μg/ml DNase I (Sigma). Cells were then plated on poly-D-lysine-coated (Sigma), eight-well glass slides (Cel-Line Associates, Newfield, NJ, USA) before fixation.

## Abbreviations

IPL, inner plexiform layer; OPL, outer plexiform layer; P, postnatal day; PKC, protein kinase C.

## Competing interests

The author(s) declare that they have no competing interests.

## Authors' contributions

EMM first noted that rod bipolars were born after cone bipolars, conducted the bipolar lineage analysis and wrote the first draft of this manuscript. AC conducted the tritiated-thymidine birthdating experiments and immunohistochemistry and contributed to the writing of the manuscript. CLC contributed to the design of the experiments, data analysis and interpretation, and to the writing of the manuscript.
